# Supplementary material for: An open-source framework for large-scale, flexible evaluation of biomedical text mining systems
Source: J Biomed Discov Collab. 2008 Jan 29;3:1. doi: 10.1186/1747-5333-3-1 (PMC2276192; doi:10.1186/1747-5333-3-1)
Supplement: Additional file 1 — Evaluation platform source code. The additional file contains the software evaluation framework discussed in this paper. The most current release can be downloaded from SourceForge.net [1]. See the accompanying README file for instructions on installation and use. Also included in the distribution is a collection of UIMA wrappers for some commonly used BioNLP tools and annotated corpora. [file 1747-5333-3-1-S1.zip › bionlp_uima_repository-1.0.1/docs/api/src-html/edu/uchsc/ccp/util/nlp/annotation/comparison/StrictSpanComparator.html]

```
001    package edu.uchsc.ccp.util.nlp.annotation.comparison;
002    
003    import java.util.List;
004    
005    import edu.uchsc.ccp.util.nlp.annotation.Span;
006    
007    /**
008     * 
009     * For "strict" comparison, the spans are required to be exactly the same in order to return 0. If they do not match exactly, then if the span start
010     * for span1 is prior to the span start for span2, then -1 is returned, 1 otherwise.
011     * 
012     * @author williamb
013     * 
014     */
015    public class StrictSpanComparator extends SpanComparator {
016    
017        /**
018         * Compare two spans using the strict (exact) match criteria.
019         */
020        @Override
021        public int compare(Span span1, Span span2) {
022            return matches(span1, span2);
023        }
024    
025        /**
026         * Compare two lists of spans using the strict (exact) match criteria.
027         */
028        @Override
029        public int compare(List<Span> spanList1, List<Span> spanList2) {
030            return matches(spanList1, spanList2);
031        }
032    
033    }
```
